# The Role of Protein Tyrosine Phosphatases in Inflammasome Activation

**DOI:** 10.3390/ijms21155481

**Published:** 2020-07-31

**Authors:** Marianne R. Spalinger, Marlene Schwarzfischer, Michael Scharl

**Affiliations:** 1 Department of Gastroenterology and Hepatology, University Hospital Zurich, 8091 Zurich, Switzerland; Marlene.Schwarzfischer@usz.ch (M.S.); Michael.Scharl@usz.ch (M.S.); 2 Zurich Center for Integrative Human Physiology, University of Zurich, 8006 Zurich, Switzerland

**Keywords:** Tyrosine phosphorylation, PTP, Inflammasome, PTPN22, PTPN2, SHP2, PTP-S2

## Abstract

Inflammasomes are multi-protein complexes that mediate the activation and secretion of the inflammatory cytokines IL-1β and IL-18. More than half a decade ago, it has been shown that the inflammasome adaptor molecule, ASC requires tyrosine phosphorylation to allow effective inflammasome assembly and sustained IL-1β/IL-18 release. This finding provided evidence that the tyrosine phosphorylation status of inflammasome components affects inflammasome assembly and that inflammasomes are subjected to regulation via kinases and phosphatases. In the subsequent years, it was reported that activation of the inflammasome receptor molecule, NLRP3, is modulated via tyrosine phosphorylation as well, and that NLRP3 de-phosphorylation at specific tyrosine residues was required for inflammasome assembly and sustained IL-1β/IL-18 release. These findings demonstrated the importance of tyrosine phosphorylation as a key modulator of inflammasome activity. Following these initial reports, additional work elucidated that the activity of several inflammasome components is dictated via their phosphorylation status. Particularly, the action of specific tyrosine kinases and phosphatases are of critical importance for the regulation of inflammasome assembly and activity. By summarizing the currently available literature on the interaction of tyrosine phosphatases with inflammasome components we here provide an overview how tyrosine phosphatases affect the activation status of inflammasomes.

## 1. Introduction

Inflammasomes are multi-protein complexes responsible for the cleavage and subsequent release of several inflammatory cytokines, including IL-1β and IL-18 [1,2]. The first step leading to the assembly of inflammasomes is activation of inflammasome receptor molecules, which subsequently form oligomers and recruit the adaptor molecule ‘apoptosis-associated speck-like protein containing a CARD’ (ASC, also known as PYCARD since it consists of a pyrin domain [PYD] and a caspase activation and recruitment domain [CARD]). Once ASC is recruited to inflammasome receptors, these receptor–ASC complexes serve as a nucleation point/scaffold for the formation of large ASC multimers called ASC-specks [1,2,3,4,5]. After their assembly, ASC-specks recruit the inactive pro-form of the inflammasome effector molecule caspase-1, which requires auto-catalysis for its activation [2]. Upon recruitment into ASC specks, pro-caspase-1 molecules are brought into close contact to each other allowing their reciprocal cleavage and activation [1,2]. Active caspase-1 finally mediates cleavage and activation of inflammasome substrates, including the pro-forms of IL-1β and IL-18, which are produced as inactive precursors to prevent their accidental release upon cell damage or during programmed cell death [1,2]. Inflammasome assembly and caspase-1 activation not only cleaves and thereby activates these pro-forms, but also mediates the cleavage and activation of gasdermin D [6,7]. Gasdermin D is a molecule of the gasdermin family that forms pores in the cell membrane to allow release of inflammasome-activated cytokines and initiates an inflammasome/caspase-1 dependent, highly pro-inflammatory form of cell death termed pyroptosis [6,7,8,9].

### 1.1. Inflammasome Receptors

To date, more than nine intracellular inflammasome-inducing receptors have been described. Each of them responds to distinct and in some cases very diverse stimuli, which typically consist of pathogen-associated molecular patterns (PAMPS), such as certain bacterial components (e.g., the bacterial cell wall product MDP, and flagellin, the main component of bacterial flagella), viral DNA, or danger/damage-associated molecular pattern (DAMP), including urate crystals, silica or other particulate molecules within the cytosol [1,2]. The most studied inflammasome-inducing receptors are pyrin, NOD-like receptor containing a pyrin domain (NLRP)1 and NLRP3, NOD-like receptor containing a CARD domain 4 (NLRC4), and absent in melanoma 2 (AIM2). However, additional inflammasomes exist [5,10], and especially the NLRP family of receptors contains multiple additional receptors that can mediate inflammasome assembly [10,11].

*NLRP1.* The first receptor that has been described to induce inflammasomes was NLRP1. The main activators of NLRP1 include bacterial products/toxins [12], such as anthrax lethal toxin [13] and the bacterial cell wall component muramyl dipeptide [14], but NLRP1 is also activated upon depletion of cytosolic ATP [15], and in general NLRP1 initiates inflammasome activation upon presence of invasive pathogens. A general mechanism of NLRP1 activation involves its proteolytic cleavage by bacterial toxins/proteases. While initially described to be mediated by anthrax lethal factor and to be restricted to the Nlrp1b isoform in mice [16], more recent studies have shown that other bacterial factors can mediate Nlrp1b cleavage [17], and that other isoforms and human NLRP1 are also activated via proteolytic cleavage [18].

*NLRP3.* NLRP3 responds to a stunningly wide range of diverse stimuli, but despite extensive investigations it is still not fully understood how NLRP3 senses such a diverse range of molecules [19]. Nevertheless, NLRP3 activation is often accompanied by mitochondrial damage and potassium efflux. Therefore, it has been suggested that rather than responding to the diverse stimuli directly, NLRP3 might somehow sense mitochondrial integrity and/or cytosolic potassium levels, which would represent the common mechanism that activates NLRP3 downstream of NLRP3-triggeing stimuli [20,21]. Furthermore, it has recently been shown that NLRP3-activating stimuli lead to disassembly of the trans-Golgi network upon which NLRP3 is recruited to the dispersed trans-Golgi network in a phosphatidylinositol-4-phosphate dependent manner, and that the disrupted trans-Golgi network serves as an assembly scaffold to mediate inflammasome complex formation [22]. However, the exact mechanism of NLRP3 activation is still unknown. While NLRP3 is critically involved in defense against microbial agents, defects in adequate NLRP3 control i.e., due to genetic mutations in NLRP3 results in auto-inflammatory diseases [23,24], and chronic NLRP3 stimulation induced by asbestos or silica drives chronic lung damage and promotes mesothelioma.

*NLRC4.* The NLRC4 inflammasome is initiated upon cytosolic presence of bacterial flagella or type 3 secretion system components. However, in contrast to other inflammasome receptor proteins, NLRC4 does not directly interact with bacterial products but serves as an adaptor for NLR apoptosis inhibitory proteins (NAIPs) [25,26,27]. Several NAIPs exist that detect the presence of a broad range of bacterial products in the cytosol [28] and activate NLRC4 upon encounter of their specific ligands [25,26,27]. Given its role in detection of bacteria, expression of NLRC4 inflammasome components in epithelial cells is required for protection against invading bacteria, and defective NLRC4 function promotes overgrowth of intestinal pathogens and intestinal dysbiosis [29,30].

*AIM2.* The AIM2 inflammasome recognizes the cytosolic presence of double-stranded DNA (dsDNA), which is typically present upon infection with bacteria or DNA viruses. In this process, AIM2 directly binds to double stranded DNA and subsequently promotes the formation of ASC multimers [31]. In contrast to other inflammasome receptors, AIM2 has not been detected within the active inflammasome complex [32], indicating an inflammasome-inducing function, but no requirement for the AIM2 receptor to provide structural stability for the formation of the inflammasome complex and the formation of ASC specks.

*Pyrin*. The inflammasome receptor pyrin reacts to the invasion of diverse pathogens into the host cell [33]. Pyrin is not directly activated by PAMPS or DAMPS, but senses changes in RhoA GTPase activity [33]. RhoA GTPase is involved in cytoskeleton rearrangement/vesicle transport and a target of many intracellular pathogens that use it to promote their uptake into the cell [33]. Thus, pyrin promotes immunity against a broad range of infectious agents.

### 1.2. Regulation of Inflammasome Activity

Given the pro-inflammatory nature of inflammasome-mediated cytokine release, its involvement in inflammatory cell death, and the potent inflammatory action of the inflammasome product IL-1β, it is not surprising that inflammasome activation is a highly regulated process and that a series of steps are required for efficient and sustained inflammasome activity [2,5,34]. As a first level of control, many of the inflammasome components, such as the inflammasome substrates, pro- IL-1β and pro-IL-18, or the inflammasome receptor NLRP3, are not expressed at all or at very low levels at steady-state, and their expression requires induction by cytokine receptor or pattern recognition receptor engagement to enable their accumulation in the cytosol [2,34,35]. Mechanisms required for transcriptional control of inflammasome components have been investigated in depth and are described elsewhere [19,34]. A second layer of control that ensures highly specific and adequate inflammasome activation is the regulation of the turnover of inflammasome receptor molecules. As an example, NLRP3 is readily ubiquitinated and subsequently degraded in proteasomes, unless this is actively suppressed upon presence of NLRP3-inducing stimuli [36,37,38]. Interestingly, ubiquitination-mediated NLRP3 inhibition is not strictly dependent on NLRP3 degradation, since deletion of the NLRP3-de-ubiquitinating molecule BRCC3 did efficiently promote NLRP3 ubiquitination and inhibited inflammasome assembly and subsequent IL-1β release, but overall NLRP3 protein levels were not affected [39]. This indicates that ubiquitination might also inhibit NLRP3 via changing its conformation and/or via impeding its interaction with activating molecules or the adaptor molecule ASC. Finally, the effect of cytokines that are released upon inflammasome activation is usually controlled via the presence of so-called decoy receptors [40]. This includes a soluble decoy receptor for IL-1, which traps IL-1β and prevents its binding to the IL-1 receptor on target cells, thus limiting IL-1β availability and bioactivity [41]. In the case of IL-18, IL-18 binding protein (IL-18BP) traps IL-18 upon release, limiting its systemic availability [42]. IL-18BP has a very high binding capacity for IL-18 [43], and a disturbed balance between IL-18 and IL-18BP expression is associated with several inflammatory diseases [44]. These mechanisms demonstrate the importance of controlled inflammasome activity to prevent exacerbated inflammatory reactions.

### 1.3. Phosphorylation as a Regulator of Inflammasome Activation

A more recently explored regulatory mechanism of inflammasome assembly/activity is the regulation of inflammasome components via their phosphorylation status. As an example, almost a decade ago, Qu et al. demonstrated that the inflammasome receptor NLRC4 is phosphorylated at Ser533 by protein kinase C upon recognition of its ligand, bacterial product-bound NAIP molecules [45]. This study indicated that phosphorylation was required for NLRC4-mediated ASC recruitment and subsequent inflammasome complex induction [45], although a newer study showed that mutation of the phosphorylation site in NLRC4 did not impair its activation [46]. Nevertheless, the description of NLRC4 phosphorylation as a critical step in promoting the ability of NLRC4 to induce inflammasome assembly, promoted the research of phosphorylation as regulatory mechanism of inflammasome activity. Subsequently several inflammasome receptors, as well as the inflammasome adaptor molecule ASC, have been described to be regulated by phosphorylation (see a recent review by Gong et al. [47] for an overview over phosphorylated inflammasome molecules).

The concept of reversible phosphorylation at specific amino acid residues to modulate the conformational stage and/or the activity of proteins has been described many decades ago [48,49,50], and nowadays it is generally accepted that phosphorylation and de-phosphorylation by kinases and phosphatases, respectively, represents one of the most common mechanisms how fast-acting intracellular signaling cascades are regulated [49,50,51]. Although phosphorylation at serine and threonine residues accounts for over 90% of all phosphorylated proteins, tyrosine phosphorylation and de-phosphorylation exerts especially important functions in the transmission of fast-acting intracellular signals and for the regulation of immune cells and inflammatory processes in the body [50,52,53]. Several inflammasome receptor molecules have potential threonine or serine phosphorylation sites [47], but tyrosine phosphorylation seems to play a pivotal role for the regulation of inflammasome assembly. Especially for NLRP3, which responds to a diverse range of danger signals [2,24], phosphorylation at tyrosine residues has been described to prevent excessive inflammasome activation [54,55]. Notably, treatment with the tyrosine kinase inhibitor, AG126, prevented DAMP- and PAMP-induced activation of caspase-1 [56], while treatment with the pan-tyrosine phosphatase inhibitor sodium orthovanadate was sufficient to induce ASC-specks, caspase-1-activation and caspase-1 dependent pyroptosis [57].

For most inflammasome receptors, potential phosphorylation sites have been reported (see ref [47] for a comprehensive overview), but for many of them, it is still unclear whether phosphorylation affects their activity. For example, it has been reported that NLRP1 and AIM2 might serve as substrates for protein kinase R (PKR) [58,59], but it is still debated whether the kinase activity of PKR is required for their activation [60,61]. With respect to the topic of the present review, however, tyrosine phosphorylation has mainly been described for the adaptor molecule ASC and for members of the NLRP family. In contrast, NLRC receptors including NLRC4 are mainly regulated by serine phosphorylation, and it is still unclear whether AIM2 is at all regulated via direct phosphorylation [61].

In this review, we summarize the effect of tyrosine phosphorylation on inflammasome components and highlight four specific tyrosine phosphatases that are involved in the regulation of inflammasomes at different stages of their activation. We will describe the influence of selected protein tyrosine phosphatases (PTP) on inflammasome activation and explore how they impact inflammatory responses in the body:

(i) PTPN22 as a direct regulator of NLRP3 activity; (ii) PTPN2 as a regulator of the phosphorylation status of the adaptor molecule ASC; (ii) SHP2 (PTPN11) as a modulator of NLRP3 inflammasome activity via regulation of mitochondrial integrity; and (iv) PTP-S2 as a regulator of caspase-1 expression.

## 2. Methodology

For this review, the PubMed database was searched for the terms “Inflammasome AND phosphorylation”, which yielded 582 results, including 39 review articles. Non-English articles were excluded from the review. All 577 articles written in English were briefly screened for those containing relevant information on tyrosine residues and whether this affected their regulation. This yielded 30 articles that were used to compile an overview on inflammasome components that that are regulated via tyrosine phosphorylation. Based on these findings, PubMed was searched for the terms “NLRP3 AND tyrosine phosphorylation”, ASC AND tyrosine phosphorylation”. The resulting search hits rendered the four tyrosine phosphatases PTPN22, PTPN2, SHP2 and PTP-S2 as relevant for the regulation for inflammasome activation. A background on the function of these four tyrosine phosphatases has been added to the description on how they regulate inflammasome activation.

## 3. Tyrosine Phosphorylation of Inflammasome Components

### 3.1. Inflammasome Receptor Molecules

Although tyrosine phosphorylation has been described for several inflammasome receptors, i.e., the NLRP molecules NLRP2, NLRP3, NLRP6, NLRP7, NLRP9, NLRP10, NLRP11, and NLRP14 [62], the effect of tyrosine phosphorylation on the activation status of most of these receptors remains largely unknown. In contrast, for NLRP3, the effect of tyrosine phosphorylation has been investigated more thoroughly and it has been described that NLRP3 tyrosine phosphorylation not only modulates its activation, but also determines its stability via promotion of its degradation [62,63]. Multiple tyrosine phosphorylation sites have been reported for NLRP3, each with slightly different outcomes regarding NLRP3 protein stability [62,63]. Phosphorylation of NLRP3 at tyrosine (Tyr)918 by the Src-family tyrosine kinase Lyn resulted in increased NLRP3 ubiquitination, suppressing its activation/promoting its proteasomal degradation [54]. However, while the kinase responsible for NLRP3 phosphorylation at Tyr918 has been determined, it is not known whether this site is controlled via tyrosine phosphatases. In turn, we have demonstrated that NLRP3 tyrosine phosphorylation at Tyr861 promotes its sequestration to autophagosomes and ultimately degradation in autophagolysosomes [64]. NLRP3 activating stimuli promoted dephosphorylation of NLRP3 at Tyr861 in a PTPN22-dependent manner, which was crucial to allow efficient and sustained NLRP3-mediated inflammasome activity. Upon loss of PTPN22, this NLRP3-stablizing effect was abrogated resulting in reduced IL-1β secretion and inefficient defeat of invading pathogens culminating in increased intestinal inflammation [55].

Additionally, several reports showed that Bruton’s tyrosine kinase (Btk) is involved in NLRP3 regulation [65]. Initially, Ito et al. reported that Btk physically interacted with ASC and NLRP3. Its pharmacological inhibition or genetic ablation impaired NLRP3 but not AIM2 inflammasome activation [65]. This was confirmed in a study by Lui et al., who validated the findings of Ito et al. in human cells [66]. Although phosphatase activity and direct interaction of Btk with NLRP3 was necessary for its ability to promote NLRP3 inflammasome assembly, Bkt did not phosphorylate NLRP3 directly, but its inflammasome-promoting effect was depending on phosphorylation of Tyr144 (Tyr146 in humans) in ASC [65]. Thus, it has been suggested that Btk might be required to promote NLRP3-ASC interaction and/or that it might be responsible for ASC tyrosine phosphorylation upon NLRP3 activation. However, the view of Btk as an inducer of NLRP3 activation has been challenged, as a recent study demonstrated that Btk inhibited the activity of protein phosphatase A2 [67], which de-phosphorylates NLRP3 at serine 5 [68]. Ser5 phosphorylation prevents accidental NLRP3 activation [68], and Mao et al. demonstrated that in the absence of strong NLRP3 inducing agents, deletion of Btk resulted in NLRP3 Ser5 dephosphorylation and subsequent inflammasome induction promoting IL-1β-dependent intestinal inflammation [67]. This clearly indicates that tyrosine kinases and phosphatases regulate inflammasome molecules in multiple ways and that a single molecule can have several functions with regards to the fine-tuning of inflammasome activity.

Furthermore, it has been demonstrated that spleen tyrosine kinase (Syk) activation is required for NLRP3 activation upon presence of *C. albicans* and other fungal species, while being dispensable for ATP or nigericin-mediated NLRP3 activation [69]. However, despite demonstrating that both NLRP3 and ASC are required for the inflammasome-promoting effect of Syk, it was not addressed which inflammasome molecule serves as the direct target of Syk, and later studies showed that Syk might actually be involved in phosphorylation of the adaptor molecule ASC, rather than targeting NLRP3 directly [70]. Acknowledging the multi-layer regulatory effect of tyrosine phosphorylation for NLRP3 activity, it will be of interest to understand whether tyrosine phosphorylation and dephosphorylation also affects the stability, activity, multimerization and adaptor recruiting capacity of other inflammasome receptors with known or putative tyrosine phosphorylation sites [47,62].

### 3.2. The Adaptor Molecule ASC

The first evidence that tyrosine phosphorylation modulates the efficacy of ASC to serve as an inflammasome adaptor and to promote inflammasome assembly downstream of several inflammasome receptor molecules was provided by a study from Hara et al. [70]. Here, the authors demonstrated that the NLRP3 and AIM2 inflammasomes required JNK and Syk activity for their full activation and ablation of either one of those two kinases interfered with sustained NLRP3- and AIM2-induced IL-1β release [70]. This defect in inflammasome activation was attributed to defective ASC-speck formation upon deletion of JNK or Syk [70]. Via mutational studies, the authors then showed that ASC phosphorylation at Tyr144 (Tyr146 in humans) was required to allow the formation of ASC-specks and sustained IL-1β release. Furthermore, ablation of JNK and Syk prevented ASC Tyr144 phosphorylation, indicating that Syk and JNK mediated phosphorylation of ASC Tyr144 is responsible to allow sustained ASC oligomerization and efficient inflammasome activation [70]. The requirement of Syk for ASC tyrosine phosphorylation and ASC-speck formation was confirmed soon after in an independent study, which postulated Syk as a target for the treatment of diseases accompanied by excessive inflammasome activity [71]. Adding to the complexity, a later study demonstrated that neither Syk nor JNK phosphorylated ASC protein directly. However, in response to inflammasome activating stimuli, they activated protein tyrosine kinase 2 (Pyk2), which seemed to be the kinase that directly phosphorylated ASC [72]. A more recent study by Mambwe et al. showed that ASC phosphorylation at Tyr144 mediated its activation, while tyrosine phosphorylation at residues Tyr60 and Tyr137 inhibited ASC-speck formation. In addition to tyrosine kinase activity of Pyk2, such tyrosine phosphatase activity was also required for full ASC activation [73]. Taken together, these findings indicate that the tyrosine phosphorylation status at specific residues determines the ability of ASC to form specks and that depending on the involved tyrosine residue, phosphorylation can have opposite effects.

## 4. Specific Tyrosine Phosphatases Involved in Controlling Inflammasome Activation

**Figure 1:**
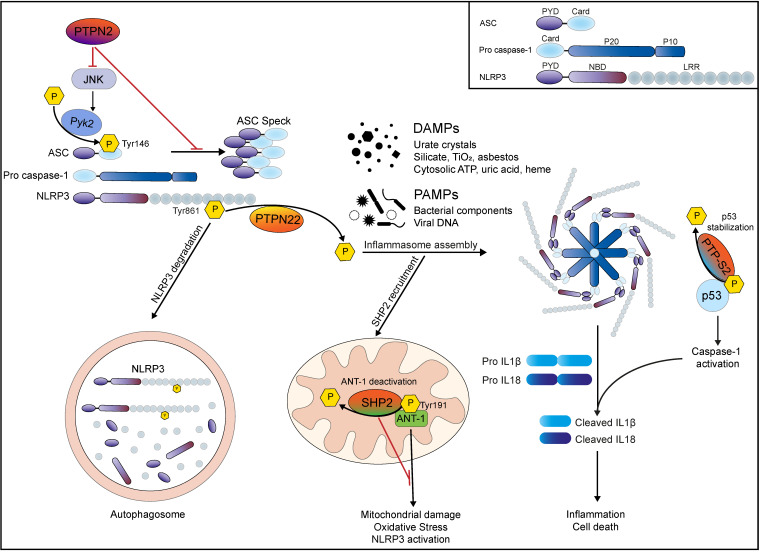
Specific tyrosine phosphatases are involved in controlling inflammasome activation: PTPN22 regulates NLRP3 activation via direct dephosphorylation. Phosphorylated NLRP3 is recruited to autophagosomes for degradation, preventing excessive inflammasome activation. PAMP or DAMP stimulation promotes PTPN22 mediated NLRP3 dephosphorylation at Tyr861, leading to its activation. Subsequent assembly of the NLRP3 inflammasome complex results in activation of caspase-1 and consequent production of IL-1β and IL-18 and inflammatory cell death. PTPN2 negatively regulates inflammasome assembly via modulation of JNK and Pyk2 activity, inhibiting ASC Speck formation, and subsequent Caspase-1 activation. Upon inflammasome induction, tyrosine phosphatase SHP2 is recruited to mitochondria where it prevents mitochondrial damage via direct dephosphorylation of ANT-1 at Tyr191. This prevents a feed-forward amplifying loop of NLRP3 activation. Nuclear PTP-S2 dephosphorylates tumor suppressor gene p53 and thereby promotes its stability. Elevated levels of p53 promote pro-caspase-1 expression and accumulation, leading to elevated caspase-1 activation and subsequent IL-1β/IL-18 activation and inflammasome-associated cell death.

In this section, we will highlight four tyrosine phosphatases (PTPN22, PTPN2, SHP2, PTP-S2) that have been described to influence inflammasome activation either directly via dephosphorylating inflammasome components, or indirectly via interfering with inflammasome activating mechanisms/pathways. An overview on the point and mode of interaction of these four tyrosine phosphatases with inflammasome components is given in Figure 1.

### 4.1. PTPN22—Allowing Efficient NLRP3 Activation via Its Dephosphorylation

PTPN22 is exclusively expressed in innate and adaptive immune cells [74,75] and controls immune responses in multiple ways. Initial studies identified PTPN22 as a potent negative regulator of T-cell receptor (TCR) signaling [76] via dephosphorylation of key signaling molecules downstream of the T-cell receptor (TCR), such as TCRζ, Lck and ζ—chain-associated protein (ZAP)70 [76,77]. By limiting the activation of naïve and effector T-cells [78,79,80,81], PTPN22 maintains immunological tolerance and homeostasis via suppression of autoreactive T-cell responses. In genome-wide associations studies (GWAS), the single nucleotide polymorphism (SNP) rs2476601 within the PTPN22 locus was identified to increase the risk for autoimmune disorders such as type 1 diabetes (T1D) [82], rheumatoid arthritis (RA) [83], systemic lupus erythematosus [84,85] and Graves’ disease [86]. Paradoxically, the very same variant has been associated with a decreased risk to develop Crohn’s disease (CD) [87]. Presence of SNP rs2476601 results in the substitution of arginine 620 (arginine 619 in mice) with a tryptophan residue resulting in an altered function protein product referred to as R620W variant. In a study to determine PTPN22 function in myeloid cells, Wang et al. described that PTPN22 controls host defense and type 1 interferon (IFNs) immune responses [88]. PTPN22 directly binds and activates TRAF3, thus promoting toll-like-receptor (TLR) signaling [88]. However, the disease-associated variant of PTPN22 failed to induce TRAF3 ubiquitination and PTPN22-variant expressing cells failed to mount efficient type I IFN responses [88]. In mouse models of experimental arthritis and DSS colitis, PTPN22 deficiency resulted in enhanced susceptibility and pronounced progression of disease, indicating that PTPN22 is required to protect from systemic and gastrointestinal inflammation [88]. Together, these data strengthened the conclusions from previous findings, suggesting that PTPN22 is a multifactorial regulator of innate and adaptive immune response [78,79,80,81,88].

While exploring the function of PTPN22 in macrophages and monocytes, we identified NLRP3 as a direct interaction partner of PTPN22 [55]. PTPN22 knockdown in THP-1 cells and loss of PTPN22 in primary murine macrophages resulted in reduced NLRP3-dependent IL-1β secretion upon stimulation with well-known NLRP3 activators. On the contrary, the autoimmunity-associated PTPN22 variant enhanced IL-1β secretion [18]. Immunoblotting revealed that PTPN22 directly interacted with NLRP3 and this interaction was enhanced upon inflammasome activation. Subsequently we demonstrated that PTPN22 promotes inflammasome activation via direct dephosphorylation of Tyr861 in NLRP3 (Figure 1). This process depended on the presence of the inflammasome adaptor molecule ASC, which was required for stabilization of NLRP3-PTPN22 protein-protein interaction [55]. Interestingly, the R619W variant of PTPN22 was found to constitutively interact with NLRP3, consequently decreasing inhibitory NLRP3 phosphorylation and thereby promote its activation [55]. A follow-up study revealed that PTPN22 regulates NLRP3 activation in an autophagy-dependent manner [64]. NLRP3 Tyr681 phosphorylation resulted in its sequestration into phagophores, diminishing inflammasome activity. NLRP3 dephosphorylation by PTPN22 protected NLRP3 from the recruitment into phagophores and thereby prevented its degradation. Notably, the autoimmunity-associated PTPN22 variant was more effective in NLRP3 dephosphorylation [64].

Mice expressing the murine R619W ortholog of the human R629W PTPN22 variant are protected in acute DSS colitis [88]. Having shown that PTPN22 expression levels are decreased in intestinal biopsies of IBD patients [89], we revealed additional immune-modulatory roles of PTPN22 in controlling autophagy and cytokine secretion [90]. Both loss of NLRP3 as well as PTPN22 resulted in aggravation of acute DSS colitis, while presence of the R619W variant protected the animals from developing intestinal inflammation [88]. Studying the molecular mechanisms, we found reduced expression levels of mature caspase-1, IL-1β and IL-18 in the lamina propria of inflamed PTPN22 KO mice but not in the epithelial cell compartment. In WT mice, DSS treatment resulted in reduced NLRP3 phosphorylation in epithelial and lamina propria cells, whereas adverse effects were observed in the lamina propria of PTPN22 KO mice [55]. Collectively, our data showed that tyrosine phosphorylation of NLRP3 suppressed its activation, and that this is directly regulated by PTPN22 [55]. The R619W PTPN22 variant, however, resulted in a gain of function with regards to NLRP3 activation [55], which resulted from enhanced substrate affinity, as well as increased phosphatase activity [55,91,92].

The NLRP3 inflammasome has been associated with the pathogenesis of different auto-inflammatory diseases such as Crohn’s disease, Alzheimer’s disease, diabetes, and atherosclerosis [24,93,94]. The autoimmunity-associated PTPN22 variant promotes NLRP3 activity, caspase-1 activation, and secretion of IL-1β and IL18, which might—at least partially—explain the pro-inflammatory character of this PTPN22 variant in T1D, RA and SLE development. In the model of acute MSU peritonitis, we demonstrated that IL-1β-mediated neutrophil infiltration was reduced in PTPN22 KO mice whereas presence of the R619W variant aggravated peritonitis [55]. Nevertheless, while promoting disease in other organs, the PTPN22 variant had protective effects in the setting of intestinal inflammation. Although surprising at a first glance, colonic NLRP3 expression is known to promote wound healing, host defense against invading bacteria [94,95,96], and promotes gut homeostasis and barrier integrity [97,98,99]. In a mouse model of acute DSS colitis, NLRP3 deficiency resulted in more severe barrier defects, erosion of the epithelium, infiltration of immune cells and alteration of the microbiome [98,100], probably due to the fact that myeloid-cell derived IL-1β in the intestine might promote wound healing and tissue repair [101].

### 4.2. PTPN2—Negative Regulation of Inflammasomes via Inhibition of ASC Phosphorylation

PTPN2 is closely related to PTPN22 and exerts prominent anti-inflammatory functions in the body. PTPN2 is expressed in all cell types and controls several pro-inflammatory mediators, including signal transducer and activator of transcription (STAT) molecules [102,103], c-Jun N-terminal kinase (JNK) and ERK [104,105,106], and thereby controls signaling cascades downstream of a multitude of cytokine and growth factor receptors [106,107,108]. PTPN2 is also responsible for the dephosphorylation of molecules downstream of the T and B cell receptor, such as Lck and Fyn [109]. Given these diverse anti-inflammatory effects, it is not surprising that genetic deletion of *PTPN2* results in generalized and uncontrolled inflammatory responses, and full-body *PTPN2* knockout mice succumb to severe systemic inflammation 3–5 weeks after birth [110]. Variants in the gene locus encoding *PTPN2* are associated with several inflammatory disorders, including IBD [111], RA [112], and diabetes [113], highlighting the important anti-inflammatory function of PTPN2. Notably, these disorders have all been associated with elevated levels of IL-1β.

With respect to inflammasome activation, PTPN2 has a dual regulatory effect: via interference with inflammatory signaling cascades [104,105], PTPN2 negatively regulates expression of inflammasome components, such as IL-1β and IL-18 while also directly affecting inflammasome assembly and subsequent release of active IL-1β and IL-18 [114]. Loss of PTPN2 results in elevated ASC tyrosine phosphorylation and subsequent ASC-multimer formation, as well as enhanced caspase-1 processing/activation and subsequent maturation and release of IL-1β and IL-18 [114]. However, although ASC activity is regulated via tyrosine phosphorylation, we found that PTPN2-mediated control of inflammasome activity was not mediated via direct interaction of PTPN2 with ASC, but that the effect of PTPN2 on ASC activity is mediated indirectly via modulation of JNK activity [114] (Figure 1). Although it has been shown JNK and Syk activity is essential for mediating ASC phosphorylation and ASC-speck formation [70,71], neither JNK nor Syk directly phosphorylate ASC, but they both promote the activity of the tyrosine phosphatase Pyk2, which is the kinase ultimately responsible for ASC tyrosine phosphorylation and subsequent promotion of its activation [72]. This shows that there are several instances upstream of ASC phosphorylation that might serve as possible points of interference to prevent inadequate and/or overshooting inflammasome activation.

Regulation of ASC-mediated inflammasome activation by PTPN2 is critical for reducing inflammatory responses to several well-described inflammasome activators, such as flagellin, particulate molecules (i.e., MSU crystals or asbestos) or bacterial toxins [114]. Defective PTPN2-mediated inflammasome control had profound effects in the setting of intestinal inflammation, where loss of PTPN2 in myeloid cells resulted in highly elevated IL-1β levels that promoted the perpetuation of inflammatory reactions, resulting in increased IL-1β-dependent intestinal inflammation [114]. Nevertheless, in the context of malignancies, such as colorectal carcinoma, increased inflammasome activity upon loss of PTPN2 had a benign effect via promotion of anti-tumor immune responses, which allowed more efficient tumor cell eradication [114]. Furthermore, increased inflammasome activation upon loss of PTPN2 might also contribute to the increased disease risk for RA and diabetes observed in *PTPN2*-variant carriers [113,115].

### 4.3. SHP-2—Interference with Mitochondrial-Damage-Associated NLRP3 Activation

NLRP3 responds to several cell damage-inducing compounds, and as mentioned above, it is still not fully understood how NLRP3 reacts to a wide range of stimuli with apparently different molecular and structural nature. However, many of these compounds promote oxidative stress and mitochondrial damage either directly or indirectly [21,116], which brings Src homology 2 (SH2) domain-containing tyrosine phosphatase-2 (SHP2, encoded by the gene *PTPN11*) into the picture of inflammasome-modulating tyrosine phosphatases. Like PTPN2, SHP2 is expressed in all tissues of the body, and it is involved in numerous signaling cascades, including growth factor and cytokine receptor induced signaling [117]. Although SHP2 is a phosphatase, it has signaling-promoting functions in several of these pathways. Specifically, SHP2 is required for efficient and sustained Ras-Raf-ERK activation downstream of growth hormone receptors, such as the epidermal growth factor (EGF) receptor, human EGF receptor 2 (her2), and Insulin-like growth factor (IGF) receptor [118,119,120]. The signaling promoting function of SHP2 seems to depend on its phosphorylation status and requires the two SH-domains present in SHP2, but its phosphatase function might be dispensable [121]. In T cells, SHP2 promotes checkpoint inhibitor molecule programmed cell death (PD)-1 induced signaling [121,122], while simultaneously dephosphorylating and inactivating ZAP70, a kinase downstream of the T cell receptor that mediates T cell receptor-induced T cell activation and proliferation. In this way SHP2 dampens T cell receptor-induced expansion of effector T cells, and allows for the immune-evasion of cancer cells [122]. Thus, SHP2 not only promotes potentially pro-oncogenic signaling pathways, but also inhibits anti-tumor T cell functions. Given its cell growth and survival promoting functions, and its involvement in dampening tumor-eradicating immune responses, SHP2 has been doubt “a proto-oncogenic tyrosine phosphatase” [119], and has emerged as a target for cancer treatment. In addition to those signaling promoting effects downstream of growth hormone receptors, SHP2 negatively regulates TLR3- and TLR4-mediated JNK, ERK and p38 activation via interfering with the TLR-adaptor molecule TRIF, and dampens TLR3 and TLR4-induced type-1 interferon and TNFα production [123]. In this way, SHP2 might also interfere with inflammasome component accumulation downstream of TLR ligation.

With regards to regulating inflammasome activation directly, Guo et al. first noticed that SHP2-deletion promoted NLRP3-mediated inflammasome activation [124], although this was not due to a direct interaction with and/or dephosphorylation of NLRP3. Instead, SHP2-deletion promoted oxidative stress and resulted in increased levels of mitochondrial damage, which was responsible for the elevated NLRP3-mediated inflammasome activity observed in SHP2-deficient cells [124]. The authors demonstrated that under normal conditions, NLRP3 activation resulted in the recruitment of SHP2 to mitochondria where it bound to and dephosphorylated ANT-1 [124]. Under physiological conditions, ANT-1 is involved in exchanging cytosolic adenosine di-phosphate with mitochondrial adenosine tri-phosphate, thus ensuring energy supply to the cell and preventing oxidative stress [125]. However, under situations of cellular stress, phosphorylated ANT-1 can promote disruption of the inner mitochondrial membrane, which leads to mitochondrial damage [126]. In this way, ANT-1 not only contributes to apoptosis initiation [127], but also potentiates NLRP3 inflammasome assembly (Figure 1) [21]. SHP-2 counteracts ANT-1-induced mitochondrial damage via direct dephosphorylation of ANT-1 [124]. In this way, SHP2 restores mitochondrial membrane integrity/prevents mitochondrial damage and thereby prevents sustained NLRP3 activation [124]. Notably, the study by Guo et al. reported that NLRP3 activation preceded mitochondrial damage and SHP2 mitochondrial translocation [124], thus mitochondrial damage potentiated NLRP3 activity, but seemed not to be responsible for its initial activation.

While macrophage-specific ablation of SHP2 promoted susceptibility to inflammasome-driven, experimental peritonitis [124], it also prevented high fat diet-induced obesity, an effect dependent on the release of IL-18 from macrophages [128]. This observation highlights the finding that depending on the context, inflammasomes can have devastating effects, but in certain settings they are protective and might have disease-preventing effects.

### 4.4. PTP-S2—Promoting Caspase-1 Overexpression via Regulation of p53

PTP-S2 is a nuclear tyrosine phosphatase that binds non-specifically to DNA within the cell nucleus. PTP-S2 is upregulated during mitosis and promotes progression through the G1 to the S phase [129,130], indicating a functional role in cell proliferation. Furthermore, overexpression of PTP-S2 induced cell death in cells that express the tumor suppressor gene p53, but not in p53 negative cells [131], indicating a negative feed-back loop to prevent excessive proliferation. p53 mediates anti-proliferative and anti-tumor effects via induction of cell cycle arrest and it induces apoptosis via upregulation of several cell-death associated molecules [132]. In resting cells, the half-life of p53 is relatively short, resulting in low cytosolic levels of p53 [132]. Upon DNA-damage or hyperproliferative signals, however, posttranscriptional modifications promote p53 stability [132], allowing for the expression of cell-death-inducing target genes. While p53 induces several genes that mediate immune-silent apoptosis [132,133], it also promotes the expression of the inflammasome effector molecule caspase-1 [134]. Via p53 dephosphorylation, PTP-S2 promoted stability, accumulation, and transcriptional activity of p53, which resulted in very high cytosolic levels of caspase-1 [135]. Despite the normally very tight regulation of caspase-1 activity, excessive cytosolic caspase-1 accumulation mediates its aberrant activation, and finally inflammatory cell death [135]. Although this regulatory mechanism has been described in the artificial setting of PTP-S2 overexpression, it demonstrates that regulation of the transcription of inflammasome components can be modulated via the activity of tyrosine phosphatases, introducing an additional mode of action how protein tyrosine phosphatases regulate inflammasome function.

## 5. Concluding Remarks

In this review, we summarized the effect of tyrosine phosphorylation on inflammasome activity and subsequent IL-1β and IL-18 release. We further described the consequence of defects in the expression of four specific protein tyrosine phosphatases that modulate inflammasome activity either directly or indirectly. As summary of these phosphatases and their action is given in Table 1. Each of these phosphatases acts in a different way to influence inflammasome activity, either directly via dephosphorylation of the inflammasome receptor molecule NLRP3, via an effect on kinases that regulate inflammasome activation, via influencing mitochondrial stability, or via modulation of the expression of the inflammasome effector molecule caspase-1. Furthermore, each of these phosphatases has fundamental roles in regulating the immune system, and regulation of inflammasome activity is just one of several functions of each of these PTPs. However, given the pro-inflammatory and potentially devastating effect of uncontrolled inflammasome activity, the effect of these PTPs on inflammasomes clearly forms an important and integral part of their impact on the immune system. Additionally, these PTPs have been implicated in diseases associated with aberrant expression of inflammasome-produced cytokines.

While focusing on NLRP3 and ASC as the inflammasome components for which tyrosine de-phosphorylation has already been explored in depth, it will be of high interest to investigate the effect of tyrosine phosphatases on other inflammasome components. Besides the well-studied effect of tyrosine phosphorylation on NLRP3, several tyrosine phosphorylation sites have been described in additional inflammasome receptors. However, to date, for most of them, neither the kinase responsible for phosphorylation has been identified, nor is it known whether these sites are targeted by tyrosine phosphatases. Therefore, the functional consequence of tyrosine phosphorylation on these receptors is still elusive. Nevertheless, with respect to those inflammasome components where tyrosine phosphorylation has been described more in depth, i.e., NLRP3 and ASC, it is obvious that the effect of tyrosine phosphorylation critically determines activation status and protein stability. Thus, tyrosine phosphatases contribute essentially to guarantee efficient, adequate, yet controlled inflammasome activation. Given the high impact of tyrosine phosphorylation on the activity of inflammasomes, studies that explore the effect of tyrosine kinases and tyrosine phosphatases on additional inflammasome components will clearly promote our understanding how inflammasomes and their potent pro-inflammatory effect in the immune system are regulated.

## 6. Take-Home Message

- Inflammasome components are subjected to regulation via tyrosine phosphorylation
- While the effect of some tyrosine phosphorylation sites on regulation of inflammasome assembly/activation is known, the effect of many putative or known tyrosine phosphorylation sites is still elusive
- NLRP3 and ASC are the most well-described inflammasome components regulated via tyrosine phosphorylation
- Tyrosine phosphorylation can have opposite effects (activation vs. inhibition)
- To date, four main tyrosine phosphatases (PTPN22, PTPN2, SHP2, PTP-S2) have been described to affect inflammasome assembly
- Future studies that assess the effect of tyrosine phosphorylation of additional inflammasome receptors will be of great relevance to understand the effect of (tyrosine) phosphorylation on inflammasomes more in depth

## Figures and Tables

**Table 1:** Overview on tyrosine phosphatases involved in controlling inflammasome activation.

Tyrosine Phosphatase	Target	Effect on Inflammasome Activation	Reference
PTPN22	NLRP3 Tyr861	De-phosphorylates NLRP3 to allow efficient inflammasome activation	[55,64]
PTPN2	JNK, Syk	Inhibits JNK/Syk mediated ASC phosphorylation and subsequent ASC speck formation	[114]
PTPN11 (SHP2)	ANT-1 in mitochondrial wall	Inhibits excessive NLRP3 activation via enhancing mitochondrial stability/preventing mitochondrial cell wall damage	[124,128]
PTP-S2	p53	Dephosphorylates p53 and thereby reduces its stability to prevent (excessive) p53-mediated *CASPASE1* gene transcription/caspase-1 protein accumulation and activation	[134,135]
